# The fibrinolytic system facilitates tumor cell migration across the blood-brain barrier in experimental melanoma brain metastasis

**DOI:** 10.1186/1471-2407-6-56

**Published:** 2006-03-09

**Authors:** George Perides, Yuzheng Zhuge, Tina Lin, Monique F Stins, Roderick T Bronson, Julian K Wu

**Affiliations:** 1 Department of Surgery, Division of Neurosurgery, Beth Israel Deaconess Medical Center, Harvard Medical School, and Department of Surgery, Tufts-New England Medical Center, Tufts University School of Medicine, Boston, MA, USA; 2 Department of Surgery, Division of Neurosurgery, Beth Israel Deaconess Medical Center, Harvard Medical School, Boston, MA, USA; 3 Department of Surgery, Division of Neurosurgery, Beth Israel Deaconess Medical Center, Harvard Medical School, Boston, MA, USA; 4 The Johns Hopkins University School of Medicine, Baltimore, MD, USA; 5 Dana-Farber-Harvard Cancer Center, Harvard Medical, Boston, MA, USA; 6 Department of Surgery, Division of Neurosurgery, Beth Israel Deaconess Medical Center, Harvard Medical School, and Department of Neurosurgery, Tufts-New England Medical Center, Tufts University School of Medicine, Boston, MA, USA

## Abstract

**Background:**

Patients with metastatic tumors to the brain have a very poor prognosis. Increased metastatic potential has been associated with the fibrinolytic system. We investigated the role of the fibrinolytic enzyme plasmin in tumor cell migration across brain endothelial cells and growth of brain metastases in an experimental metastatic melanoma model.

**Methods:**

Metastatic tumors to the brain were established by direct injection into the striatum or by intracarotid injection of B16F10 mouse melanoma cells in C57Bl mice. The role of plasminogen in the ability of human melanoma cells to cross a human blood-brain barrier model was studied on a transwell system.

**Results:**

Wild type mice treated with the plasmin inhibitor epsilon-aminocaproic acid (EACA) and *plg*^-/- ^mice developed smaller tumors and survived longer than untreated wild type mice. Tumors metastasized to the brain of wild type mice treated with EACA and *plg*^-/- ^less efficiently than in untreated wild type mice. No difference was observed in the tumor growth in any of the three groups of mice. Human melanoma cells were able to cross the human blood-brain barrier model in a plasmin dependent manner.

**Conclusion:**

Plasmin facilitates the development of tumor metastasis to the brain. Inhibition of the fibrinolytic system could be considered as means to prevent tumor metastasis to the brain.

## Background

There are about 150,000 metastatic brain tumors diagnosed annually compared to 17,000 primary brain tumors in the United States and the incidence of CNS metastases is steadily increasing [1-4]. While advances in cancer therapy have translated into longer survival for patients with extraneural disease, these advances have, ironically, increased the number of patients who develop CNS metastases. Despite decades of intensive investigation, and increasing success in treating extraneural cancer, the median survival for patients with CNS metastases from systemic cancer is only 6-10 months [5]. In patients with CNS metastasis, neurologic disease is the primary cause of disability in approximately half of the cases when treated with whole brain radiation and a third of the cases if treated with surgery and whole brain radiation [6]. Therefore, many patients experience significant disabilities in the final weeks of life. CNS metastases are also an important cause of disease relapse following high-dose chemotherapy with bone marrow or stem cell support [7] and a common reason for exclusion of patients from high-dose chemotherapy protocols because of poor penetration of drugs into the CNS. For these reasons, strategies for preventing CNS metastases and growth from systemic cancer are vitally important.

Successful metastasis, depends on extracellular matrix remodeling which in turn depends on proteolytic enzymes [8]. Plasmin, a key component of the fibrinolytic system is a promiscuous serine protease which directly digests collagen, laminin, fibronectin, and proteoglycans in the extracellular matrix and indirectly by activating other matrixolytic enzymes (e.g. matrix metalloproteinases). Plasmin is generated after activation of plasminogen by urokinase- and tissue-type plasminogen activator (uPA and tPA) [9,10]. uPA and tPA in turn can be inhibited by the plasminogen activator inhibitors -1 and -2. Melanoma is an excellent model to study the role of the fibrinolytic system since it has a high propensity for brain metastasis. Forty eight percent of patients with malignant melanoma develop brain metastasis [11]. The central nervous system appears to be a sanctuary for cancer cells in patients being treated with most systemic cytotoxic chemotherapies and immunotherapies. Consequently, as the systemic therapy for metastatic melanoma improves, CNS relapse is becoming more frequent and a major barrier to long term survival. The median survival of patients with melanoma metastatic to the central nervous system is less than six months.

## Methods

### Cell culture

B16F10 mouse melanoma cells and SK-Mel human melanoma cells were cultured in Dulbecco's modified Eagle's medium (DMEM) and 10% fetal calf serum (FCS). Human brain microvascular endothelial cells (BMEC) were prepared as described previously [12]. The cells were maintained in DMEM:Ham's F12 (1:1) containing 10% FCS (Invitrogen, Carlsbad, CA) and 10% Nu serum (BioWhittaker, Inc. Walkersville, MD).

### Cell labeling

#### Radioactive labeling of B16F10 mouse melanoma cells

Half a million B16F10 cells were plated in 100 mm petri dishes and left to grow for 48 hours in DMEM/F12 containing 10% fetal calf serum. The medium was removed and serum starved for 24 hours after which the medium was exchanged and 10 ml of DMEM/F12 containing 1μCi of ^125 ^I-deoxyuridine (Perkin Elmer Boston, MA) was added. The next day the medium was removed and the cells washed with DMEM/F12 and used for further experiments.

#### Labeling with Cell Tracker Orange™ (Molecular Probes, Eugene, OR)

Cells were plated in 100 mm ø petri dishes to reach confluence. They were then washed with DMEM/F12 and 5 ml DMEM/F12 containing 10 μM Cell Tracker Orange™ and incubated for 40 min at 37°C in a 5% CO_2 _and 100% humidity. Cells were washed with DMEM/F12 and incubated for another 40 min. to allow the Cell Tracker Orange™ to be incorporated.

### In vitro blood-brain barrier model

To create an in vitro model of the human blood-brain barrier (BBB), 50,000 human BMEC were seeded on top of semipermeable Transwell™ polycarbonate tissue culture inserts (pore size 8μm, 6.5 mm ø) (Corning Costar Corp. Corning, NY). After 3 days the human BMEC formed a continuous monolayer [13]. At this point the transendothelial electrical resistance (TEER) of this monolayer was usually around 300-500 Ohm/cm^2^. The TEER was maintained at this level for the next 5-7 days. The TEER then declined and soon thereafter cells appeared to detach from the monolayer. For our experiments only monolayers with TEER greater than 300 Ohm/cm^2 ^were used.

### Melanoma cell migration across BMEC

SK-Mel cells labeled with Cell Tracker Orange™, were plated on the top of the BMEC monolayer in DMEM/F12. As a chemo-attractant DMEM/F12 containing 10% FCS was added to the bottom chamber. The next day the inserts were examined for the presence and the location of the cancer cells. The top site of the insert was cleaned with a Q-tip swab and the remaining cells photographed and counted. To ensure that these cells had crossed the BMEC monolayer the bottom site of the insert was also cleaned with a Q-tip. Cells that were not removed by the Q-tip were not considered as having successfully crossed the BMEC monolayer.

### Animal tumor models

Plasminogen heterozygous mice in a C57Bl background were a kind gift from Dr. Carmeliet (Vlaams Interuniversitair Institut voor Biotechnologie, Leuven, Belgium) [14]. They were further backcrossed 6 times to commercially available C57Bl (Jackson Laboratories, Bar Harbor, ME) and then intercrossed to generate wild type, heterozygous and homozygous plasminogen deficient mice. Plasminogen deficient mice have a limited lifespan (6-8 months) and are not efficient breeders and the colony is maintained by crossing the heterozygous mice. For our experiments 8-12 week old wild type and homozygous deficient mice were used while the heterozygous mice were kept to propagate the colony.

### Intracarotid injection of tumor cells

To simulate hematogenous metastases to the brain we employed an intracarotid tumor injection model in the C57Bl mouse. Our intracarotid injection model is similar to the one described by Schackert and Fidler [15]. Modifications include a) we used a 30 G needle to inject the cells instead of a glass needle, and b) we sutured the site of injection in the carotid artery to restore the blood circulation. C57B mice were anesthetized with 50 mg/kg xylazine 5 mg/kg ketamine cocktail. Mice were prepped with betadine and all operation took place under the dissecting microscope. A 1 cm incision in the neck was performed and the left common carotid artery was exposed. The external carotid artery was temporarily occluded with a 6-0 prolene and the common carotid artery was temporarily ligated with a microclip (George Tiemann, Hauppauge, NY). A 30 G needle connected to PE-10 tubing (Becton Dickinson, San Diego, CA) was inserted into the lumen of the common carotid artery. The 5 μl cell suspension was then injected and chased with 40 μl of Hanks balanced salt solution over a period of 1 minute. Distal to the needle a microclip was placed and the needle removed. The artery was sutured with a 10-0 prolene (Johnson & Johnson, Sommerville, NJ) and the microclips removed to re-establish the blood flow. In most cases no bleeding was observed but if there was some it could be stopped with gentle pressure on the arteriotomy for 30-60 seconds. Mice were returned to their cage with unlimited access to food and water. More than 90% of the mice recovered completely after the surgery and without any neurological signs.

### Intracranial injection of tumor cells

Anesthetized mice were placed in a stereotactic frame Kopf (Tujunga CA) prepped with betadine and a 0.5 cm incision was performed along the midline of the skull. Two mm left of the bregma a burr hole was drilled and tumor cells were injected 3 mm deep in the striatum over the course of 5 min. Cells were resuspended in DMEM/F12 at a concentration of 2 × 10^8^/ml and 5 μl were injected at a 1 μl per minute rate. After injection the needle was left in the striatum 5 min and then it was removed. The skin was sutured and the animals returned to their cage with unlimited access to food and water.

## Results

### Fate of intracarotid injected mouse melanoma cells

**Figure 1:**
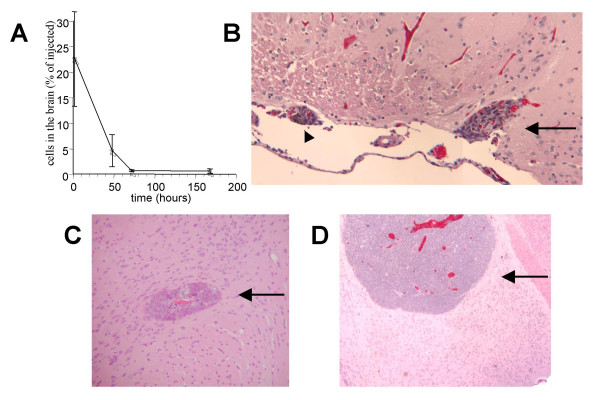
**Metastatic brain tumor model. **A) One hundred thousand ^125^I-deoxyuridine-labeled B16F10 cells were injected in the left internal carotid artery. Mice were sacrificed after 1, 24, and 48 hours, 3 days and 7 days. The left hemisphere was removed and the amount of radioactivity was measured by means of the ICN gamma-counter to determine the number of cells remaining in the brain. Note the rapid decline of radioactivity in the brain that remained stable after 72 hours. Hematoxylin-eosin staining of melanoma tumors developing in the brain after intracarotid injection (B-D). B) B16F10 cells lodged in a blood vessel (arrowhead) and cells invading the brain (arrow). C) B16F10 cells (arrow) growing in the brain parenchyma. D) Large tumor growing in the brain parenchyma (arrow).

To determine the fate of the injected cells we labeled B16F10 cells with ^125^I-deoxyuridine. ^125^I-deoxyuridine is incorporated in the DNA of the B16F10 cells and is not taken up by other mouse cells after injection [16]. It is then secreted in the urine. As reported earlier [17] the number of cells that remains in the brain rapidly decreases after the injection over the course of hours to days. Only a small percentage (0.69+/- 0.23%) was found in the brain after 72 hours (Fig. 1A). These cells remained stable over the course of ten days suggesting that the detected radioactivity in the brain was from living cells that gave rise to the subsequent tumors. The organs with the next highest level of radioactivity were the lungs followed by the kidney and the spleen. The levels of radioactivity in these organs however, continued to decline over the course of the next 10 days indicating that it represents dying cells. This interpretation was further supported by the absence of tumors in any of these organs two weeks after surgery. The only tumors identified were in the brain. On occasion, tumors were seen growing in the neck of these animals at the site of injection indicating that some cells had either leaked during the procedure or adhered to the carotid artery. Histologic examination of the hematoxylin-eosin stained brain section from wild type mice indicated that the cells were lodged in the blood vessels (Fig. 1B). From the blood circulation the cells extravasated into the brain (Fig. 1B, C). Once there, the tumors started growing into large masses (Fig. 1D) that often occupied large areas of the brain frequently as much as 25% of the brain volume depending on the time of incubation and the number of cells injected.

### Role of plasminogen in metastatic tumors to the brain

**Figure 2:**
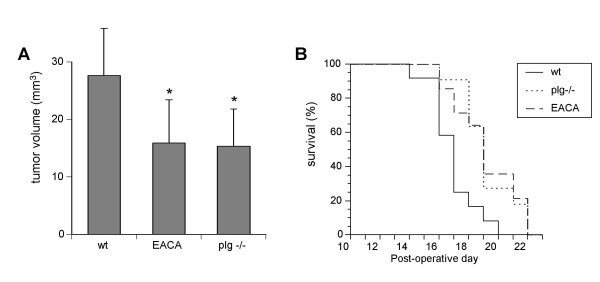
**Effect of plasminogen in tumor metastasis. **Metastatic brain tumors were established by injecting 10^6 ^B16F10 cells in the left internal carotid artery of wild type wild type C57Bl (wt), plasminogen deficient (plg-/-) and wild type mice treated with EACA (EACA). Fourteen days after tumor cell injection the mice were sacrificed the brains removed fixed and stained with hematoxylin eosin. The areas of the tumors at 200 μm intervals were measured and the total volume of the tumor determined. Note the reduced tumor size in mice treated with EACA and plg-/- mice compared to wild type mice. B) Survival graph of 12 wild type mice (wt), 11 *plg*^-/- ^mice (plg-/-) and 7 wild type mice treated with EACA (EACA) injected with 10^6 ^B16F10 cells. Note the increased survival of *plg*^-/- ^and wild type mice treated with EACA compared to untreated wild type mice (p < 0.03)

With the knowledge that we could reproducibly obtain brain metastases in our intracarotid mouse model we then set out to determine whether the fibrinolytic system plays a role in the tumor metastasis to the brain. We injected 100,000 B16F10 cells in the carotid artery of wild type mice and mice lacking plasminogen (*plg*^-/-^). After 14 days all mice (wild type, and *plg*^-/-^) were sacrificed and the brains removed and fixed in 4% paraformaldehyde overnight. The brains were cut in 2 mm slices embedded in paraffin and sections were obtained every 200 μm intervals. Sections were stained with hematoxylin and eosin and photographed with a SPOT camera mounted on a Nikon E800 eclipse microscope. The tumor volume was calculated by integrating the tumor area with the length of the tumor. The tumor volume in *plg*^-/- ^mice was 44.5% smaller than the tumor volume found in the brains of wild type mice (Fig. 2A). To ensure that the difference in survival was not due to genetic abnormalities that occurred with the genetic manipulations to generate the knockout animals, wild type mice were also treated with the plasmin inhibitor ε-aminocaproic acid (EACA). Two and a half percent of EACA was added in the water of the mice two days prior to the operation and it was changed daily. At this concentration the EACA levels in the blood is 275-300 μg/ml that completely inhibits the conversion of plasminogen to plasmin [18,19]. The tumor volume was 42.4% smaller than the tumor seen in wild type mice which would add support to our finding that plasminogen most likely participate in tumor metastasis to the brain of the *plg*^-/- ^mice (Fig. 2A).

We then determined whether the reduction in tumor burden translates into increased survival. In these experiments the 12 wild type mice died, on average after 16.9+/-1.6 days compared to the 11 *plg*^-/- ^mice that died on average after 19.2+/-1.9 days. The 7 EACA treated wild type mice also survived longer (18.8+/-1.3. days) than the untreated wild type mice. Kaplan-Meyer analysis showed these differences are statistically significant (log rank test, p < 0.03) (Fig. 2B).

**Figure 3:**
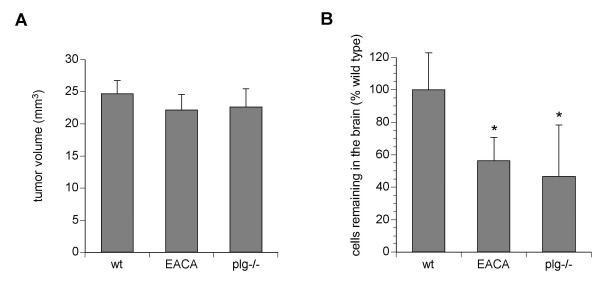
**Plasminogen facilitates the process of metastasis but not tumor growth in the brain. **A) Ten thousand B16F10 cells were injected in the striatum of 5   wild-type mice (control), 5 *plg^-/-^* mice (plg-/-) and 5 wild type mice   treated with EACA (EACA). The mice were sacrificed 12 days after tumor   cell injection and the brains removed to determine the size of the   growing tumor (bars show the standard deviation). No statistically   significant difference between the three groups was observed. B)   Wild-type mice (wt),*plg^-/-^* mice and wild type mice treated with EACA   (EACA) were injected with 100,000 125I-deoxyuridine labeled B16F10   cells in the internal carotid artery. They were sacrificed 3 days later   and their brains removed to determine the amount of radioactivity and   compared to the total amount radioactivity injected in these mice. The   bars represent the average of six mice for each group (bars show the   standard deviation). The amount of radioactivity found in the wild type   mice was considered as 100% *p<0.01.

The observed differences in tumor size and mouse survival could be due to the effects of the fibrinolytic system in tumor growth and/or the process of metastasis. We first tested whether the fibrinolytic system affects tumor growth directly in the brain by bypassing the blood-brain barrier. We injected 100,000 B16F10 cells directly in the striatum of wild type mice, wild type mice treated with EACA and *plg*^-/- ^mice. Twelve days after tumor cell implantation the mice were sacrificed and the brains removed to determine the volume of the growing tumors. No statistically significant difference in the size of these tumors was observed (Fig. 3A).

To determine whether the difference in tumor volume was due to the difference in the number of tumor cells metastasizing to the brain we labeled B16F10 cells with^125^I-iodo-2'-deoxyuridine and injected them into the internal carotid artery. Seventy two hours after injection mice were sacrificed, the brains removed and the radioactivity measured to determine the number of cells. Plasminogen deficient (*plg*^-/-^) mice had 0.30%+/-0.1 of the initially injected cells in the brain compared to 0.69% in wild type mice a 53.3+/-14.4% reduction (Fig. 3B). Similarly, EACA treated mice contained 0.38%+/- 0.22 of the injected cells, 46.6% fewer cells compared to untreated wild type mice. Taken together these results suggest that the fibrinolytic system in particular plasmin is important in the establishment of the metastasis to the brain but not the growth of the tumor in the brain once it gets there.

### Melanoma cells crossing the blood-brain barrier model

**Figure 4:**
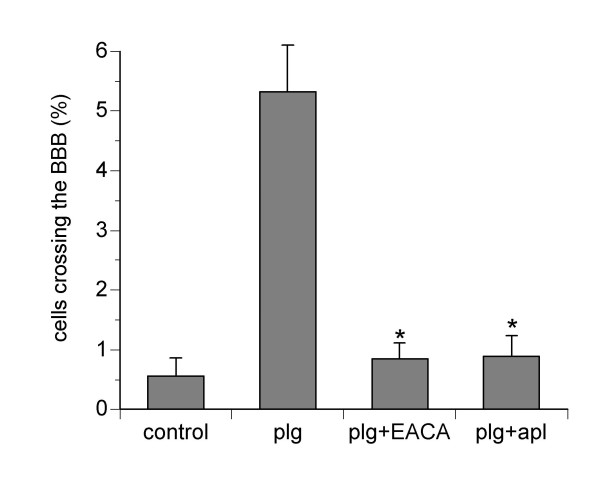
**Cancer cell transversal of the in vitro blood-brain barrier model. **50,000 BMEC were plated on 6.5 mm ø transwells coated with collagen and 8.0 μm pore size in DMEM/F12/10%FCS/10%Nu serum. After 3 days the cells had grown to confluence and the medium was removed and replaced with DMEM/F12. After two more days 10,000 human SK-melanoma cells (SK-Mel) labeled with Cell-Tracker Orange™ were added. These co-cultures were further incubated in the absence of exogenous plasminogen (control), in the presence of 1 μg/ml plasminogen (plg) and in the presence of plasminogen and EACA (EACA). The results are the average of three experiments performed in duplicate. Error bars show standard deviation. *p < 0.01.

To further understand the role of the fibrinolytic system in tumor cell invasion of the brain we employed an in vitro blood-brain barrier model formed by human brain microvascular endothelial cells (BMEC) [12,20]. The BMEC are positive for FVIII-Rag, carbonic anhydrase IV, *Ulex europeus *agglutinin I and take up acetylated low-density lipoprotein (AcLDL) demonstrating their endothelial origin and also express gamma glutamyl transpeptidase, demonstrating their brain origin [12,20]. Brain endothelial cells with these phenotypic expression were previously immortalized by transfection with the SV40-large T-antigen [20]. To determine whether tumor cells were able to cross the blood-brain barrier model they were first labeled with Cell Tracker Orange (Molecular Probes, Eugene, OR). Human SK-Mel cells were placed on top of a monolayer formed by BMEC in the presence or absence of plasminogen and the presence or absence of the plasminogen inhibitor EACA and α_2_-antiplasmin. At the end of the experiment the cells were photographed and counted. In the absence of plasminogen virtually no cells crossed the BBB model (0.56+/-0.3%). However, by adding human plasminogen a 9-fold increase (5.32+/-0.78%) in the number of cells crossing the BBB model was observed. By adding the plasminogen inhibitor EACA or α_2_-antiplasmin in addition to plasminogen we saw strong inhibition of the plasminogen-dependent inhibition of tumor cell migration across the BBB (0.85+/-0.26% and 0.89+/-0.32%, respectively) indicating that plasminogen is necessary for crossing of the BBB model (Fig. 4).

## Discussion

Since the first correlation between cancer and plasminogen activation was drawn more than 25 years ago [21] there has been more than 100 reports on the prognostic value of uPA, tPA and the plasminogen activator inhibitor -1 (PAI-1) (reviewed in [22-24]. Several studies on experimental tumor models have been published suggesting that inhibition of fibrinolytic enzymes can inhibit tumor growth and metastasis [25-27]. EACA, a plasmin inhibitor, reduced tumor growth by 37% in a xenograft tumor model of glioma [18]. A synthetic inhibitor trans-4-amino ethylcyclohexanecarbonyl-tyr-(O-Pic)-octylamide inhibited human lung metastasis in a xenograft mouse model [28]. Prostate carcinoma metastasis in a mouse model was prevented by a synthetic urokinase inhibitor [29]. A study using overexpression of uPA receptor in breast carcinoma showed increased invasion and metastasis suggesting a role of uPA in tumor growth [30]. Melanoma cells express uPA and uPA receptor which may facilitate activation plasmin [31]. In a survey of skin cancers the levels of uPA correlated with the malignancy of the tumors with malignant melanoma containing the highest amounts of uPA, followed by squamous cell carcinoma and basal cell carcinoma containing the least amount of uPA [32]. Inhibition of plasminogen and plasminogen activators reduced melanoma cell migration in an *in vitro *model [33]. Melanoma cells transfected with plasminogen activation inhibitor-1 developed fewer metastases than not transfected cells in a lung metastasis model [34] and in an ocular melanoma tumor model [35].

Brain metastases from systemic cancers are increasing despite advances in systemic treatments. In an attempt to dissect the underlying mechanisms for brain metastases we injected melanoma tumor cells directly into the carotid arteries of mice to establish a model for hematogenous metastases to the CNS. By manipulating the presence or absence of plasminogen with plasminogen knockout mice we demonstrated that in the absence of the expression of plasminogen the metastatic tumors were smaller and the survival of the corresponding mice were significantly longer. When we pretreated the wild type mice with EACA, a plasminogen inhibitor, prior to intracarotid injection of melanoma cells the brain metastases were also smaller and these animals survived longer similar to the plasminogen knockout mice. The smaller tumors in the EACA treated and plasminogen knockout mice could be due to inhibition of tumor growth in the brain once the melanoma cells reached there or inhibition of tumor cells moving across the blood-brain barrier. Our experiments with direct brain injections of tumor cells in the plasminogen knockout mice and EACA treated wild type mice showed no reduction of tumor size compared to the tumors in wild type mice. These results would suggest that plasmin is important in the migration of tumor cells across the blood-brain barrier into the brain parenchyma and not in the growth of the tumor once it is established in the brain. Plasmin can modulate cell adhesion by directly digesting tenascin as it has been shown in lymphocytes [36]. It can promote cell motility by regulating the interaction between vitronectin and uPA [37]. It can facilitate cell migration across the blood-brain barrier by directly digesting fibronectin and laminin [38,39]. In the brain parenchyma plasmin can interact and digest the L1-cell adhesion molecules to disrupt cell adhesion [40]. In addition, to these direct effects, plasmin activates matrix metalloproteinases that in turn digest other extracellular matrix proteins or modulate gene expression of other genes [41]. To determine whether migration of tumor cells across the BBB is dependent on plasmin we utilized an in vitro BBB model using human brain microvascular endothelial cells (BMEC). These experiments demonstrated that the migration of human melanoma cells across the BMEC was dependent on plasmin. With inhibition of plasmin significantly fewer melanoma cells cross the BMEC. However, these in vitro studies do not take into account the possibility that tumor cells can simply get lodged in the capillaries and start proliferating there. Once they reach a critical mass the capillaries may simply break allowing the cells to enter the brain parenchyma.

## Conclusion

Our experiments suggest that the fibrinolytic system is involved in facilitating melanoma cells cross of the blood-brain barrier, but the minimal increase in animal survival, suggests that other enzymes may be involved. Studies suggest that matrix metalloproteinases (MMPs) and a disintegrin and metalloproteinase family of proteolytic enzymes (ADAMTS) may also play a role in the development of metastases [42,43]. In fact, inhibition of matrix metalloproteinases, leads to restoration of the blood-brain barrier in an experimental autoimmune encephalomyelitis [44]. Further understanding of the mechanisms by which systemic cancers metastasize to the brain will enable us to devise novel methods to inhibit brain metastasis across the blood-brain barrier and improved survival for patients.

## List of abbreviations

blood-brain barrier, BBB; brain microvascular endothelial cells, BMEC; central nervous system, CNS; Dulbecco's modified Eagle's medium, DMEM; epsilon-aminocaproic acid, EACA; fetal calf serum, FCS; plasminogen, plg; urokinase-type plasminogen activator, uPA; tissue-type plasminogen activator, tPA.

## Competing interests

The author(s) declare that they have no competing interests.

## Authors' contributions

GP designed the studies, performed intracranial injections and migration experiments, analyzed the data and prepared the manuscript. YZ performed intracarotid injections and migration experiments, TL performed intracarotid injections. MS developed the blood-brain barrier model, RB analyzed the histology slides, JKW analyzed the data and prepared the manuscript. All authors read and approved the manuscript.

## Pre-publication history

The pre-publication history for this paper can be accessed here:
